# miRNA-200a/c as potential biomarker in epithelial ovarian cancer (EOC): evidence based on miRNA meta-signature and clinical investigations

**DOI:** 10.18632/oncotarget.13154

**Published:** 2016-11-07

**Authors:** Yue Teng, Xuan Su, Xing Zhang, Yan Zhang, Chen Li, Wenquan Niu, Chang Liu, Kai Qu

**Affiliations:** ^1^ Department of Obstetrics and Gynecology, The First Affiliated Hospital of Xi'an Jiaotong University, Xi'an 710061, China; ^2^ Department of Head and Neck of Sun Yat-sen University Cancer Center, State Key Laboratory of Oncology in South China, Collaborative Innovation Center for Cancer Medicine, Guangzhou 510060, China; ^3^ Department of Hepatobiliary Surgery, The First Affiliated Hospital of Xi'an Jiaotong University, Xi'an 710061, China; ^4^ Center for Translational Medicine, The First Affiliated Hospital of Xi'an Jiaotong University, Xi'an 710061, China; ^5^ State Key Laboratory of Medical Genomics, Ruijin Hospital, Shanghai Jiaotong University School of Medicine, Shanghai 200025, China

**Keywords:** miRNA-200a/c, epithelial ovarian cancer (EOC), Robust Rank Aggregation

## Abstract

Extensive effort has been put on miRNA expression signatures in epithelial ovarian cancer (EOC). Unfortunately, consistent conclusion rarely yielded from diverse studies, mainly due to the high inter-lab variability and small sample sizes. To overcome above limitations, an integrated analysis of miRNA expression signature was performed by employing Robust Rank Aggregation (RRA) method. Diagnostic analysis, Kaplan-Meier survival curves and pathway enrichment analysis were used to investigate the clinical values and biological functions of meta-signature miRNAs. A total of 519 EOC and 248 noncancerous samples were included. Seven mostly dysregulated miRNAs were identified by RRA method and two miRNAs (miR-200a-3p and miR-200c-3p) remained statistically significant after Bonferroni-correction. Diagnostic meta-analysis showed reliable diagnostic capacity of miR-200a-3p (with a pooled sensitivity of 0.84 and specificity of 0.83) and miR-200c-3p (with a pooled sensitivity of 0.75 and specificity of 0.66) for EOC. Pathway enrichment analysis and expression correlation analysis suggested miR-200a/c might contribute EOC progression by affecting cellular adhesion process. Kaplan-Meier survival analysis based on two independent cohorts revealed a strong association between miR-200a/c and overall survival in EOC patients. miR-200a/c was identified as the mostly dysregulated miRNAs in EOC and might be novel diagnostic and prognostic biomarkers for patients with EOC.

## INTRODUCTION

Epithelial ovarian cancer (EOC) accounts for 25% of all malignancies affecting the female genital tract and is the most lethal gynecological malignancy, accounting for 4.2 % of all cancer-related deaths in women. Most EOC patients are diagnosed at late stages, leaving little chance for survival due to the lack of effective treatments [1, 2]. During the past century, incidence of EOC has been slowly yet steadily increasing, while development of more effective treatment has lagged behind, leading to little, if not none, improvement in overall survival. Current standard treatment for EOC includes a combination of surgical resection and chemotherapy, which acts efficiently as initial treatment. However, most EOC patients recur after a few years and turn to be resistant to existing treatments [3, 4]. Despite the use of aggressive treatment, recurrence is frequently seen among EOC patients, and cancerous metastasis is one of the predominant causes of mortality. Therefore, exploration of novel biomarkers for early diagnosis, prognosis prediction, and effective therapies will definitely contribute to current EOC treatment and management.

As a group of short endogenously noncoding RNA molecules, microRNAs (miRNAs) are drawing increasing attention for their versatile activities in various physiological and pathological processes. By imperfect complementary sequence pairing between miRNA seed region and the 3’-untranslated region (UTR) of target genes, miRNAs negatively regulate target genes by either mRNA degradation or translational repression, thus directly or indirectly affecting almost all cellular pathways [5, 6]. Recently, rapid technological advances in platforms for high-throughput miRNA profiling have generated profiles and signatures of miRNAs associated with various cancer types [7, 8]. A group of differentially expressed miRNAs stood out as potential biomarkers for diagnostic, prognostic and therapeutic applications in cancers, including ovarian cancer [9, 10]. Unfortunately, no consistent conclusion has ever been made from miRNAs profiling studies on ovarian cancer. Confounding factors may include, and are not limited to, employment of different detection platforms, small sample size, inconsistent annotation of miRNAs, ongoing discovery of novel miRNAs, discrepancy in clinico-pathological characteristics and author defined cut-off criteria of differentially expressed miRNAs, and application of different statistical methods [11–14].

To overcome the limitations in current researches, we integrated these results by performing a meta-analysis applying the recently published robust rank aggregation (RRA) method [7], followed by pathway analysis, to identify miRNA deregulation in ovarian cancer and the pathways that key miRNAs may affect [15]. The RRA approach has been specifically designed for comparison of several ranked gene lists and identification of commonly overlapping genes. This method is a suitable and effective solution for identification of statistically significant miRNA meta-signature and is particularly useful when input experiments are performed by different technological platforms cover different sets of genes and full rankings of miRNAs are not available [16]. Identification of miRNA meta-signature and exploration of involved pathways would provide potential targets for further experimental studies of ovarian cancer development. Furthermore, we validated the most consistently dysregulated miRNAs in public data atlas. Those miRNAs extracted from screening and validation will probably be eligible markers for the early detection and prognosis prediction of ovarian cancer.

## RESULTS

### Characteristics of included studies

According to the search criteria, a total of 14 independent full-text studies retrieved from public databases were used to build the EOC miRNA expression profiling datasets [17–29]. The basic characteristics of 14 studies, including first author, year of publication, ethnicity, country, study period, sample number, cancer subtype, sample source, detection methods, number of detected miRNA, and cut-off criteria were listed in Table 1. Our pooled dataset included a total of 519 cancer and 248 noncancerous tissue samples across the studies. Various microarray platforms were used in the studies and the numbers of miRNA probes assayed ranged from 85 to 2064. Expansion of studied miRnome from 2007 to 2015 is reflected by the wider distribution of the differentially expressed miRNAs in later datasets (Table 1). The number of significantly dysregulated miRNAs varies greatly across the studies (range from 2 to 77 miRNAs). The overall rank matrixes of normalized upregulated and downregulated miRNAs were separately listed and analyzed in the following analysis.

**Table 1:** Characteristics of studies included for meta-analysis of miRNA expression in ovarian cancer

Author (year)	Country	Ethnicity	Period	No. of sample (T/N)	Cancer subtype	Sample source	Method	Total miRNAs	Cut-off criteria
Iorio(2007) [17]	USA	Caucasian	NA	69/15	31 serous, 8 endometrioid, 4 clear cell, 9 poorly differentiated, 1 mucinous carcinoma.	Frozen tissue	Microarray	235	*p*<0.01 and FC>3
Nam(2008) [18]	Korea	Asian	2000.12-2003.9	20/8	20 serous ovarian carcinoma	Frozen tissue	Microarray	314	FC>2
Dahiya (2008) [19]	USA	Caucasian	NA	34/1^a^	31 serous, 2 clear cell, 1 borderline carcinoma	Frozen tissue	Microarray	462	FC>2
Yang (2008) [20]	USA	Caucasian	NA	10/10^a^	10 serous ovarian carcinomas	Frozen tissue	Microarray	515	FC>1
Wyman (2009) [21]	USA	Caucasian	NA	33/4^a^	19 serous, 4 clear cell 10 endometrioid carcinoma	Frozen tissue	Sequencing	498	FC>4
Resnick (2009) [22]	USA	Caucasian	NA	28/15	17 serous, 6 clear cell, 3 endometrioid, 2 mucinous carcinoma	Frozen tissue	Microarray	365	FC>1.5
Kim (2010) [23]	Korea	Asian	NA	54/49^b^	29 serous, 11 mucinous, 7 endometrioid, 7 clear cell carcinoma	Frozen tissue	Microarray	739	*p*<0.01
Elgaaen (2014)a [24]	Norway	Caucasian	2003-2012	35/9	35 high-grade serous carcinoma	Frozen tissue	Microarray	1105	*p*<0.01
Elgaaen (2014)b [24]	Norway	Caucasian	2003-2012	19/9	19 clear cell carcinoma	Frozen tissue	Microarray	1105	*p*<0.01
Dong (2014) [25]	China	Asian	2008.4~2012.7	5/5	5 high-grade serous carcinoma	Frozen tissue	Microarray	2064	FC>2 and *p*<0.05
LI (2014) [26]	China	Asian	NA	100/50	100 serous carcinoma	PPFE	Microarray	739	FC>2
Shapira (2014) [27]	USA	Caucasian	NA	42/36	42 serous carcinoma	Plasma	Microarray	754	FC>2
Wang (2014) [28]	China	Asian	NA	48/15	29 serous, 6 mixed epithelial, 6 endometrioid, 1 adenocarcinoma, 4 clear cells, 2 mucinous carcinoma	Frozen tissue	High throughput PCR	1757	FC>1.5
Ibrahim (2015) [29]	Malaysia	Asian	2006-2013	22/22	22 serous ovarian carcinoma	Frozen tissue	Microarray	85	FC>2

a Immortalized human ovarian surface epithelial cells were used as control.

b Benign tumors were used as control.

FFPE, formalin-fixed paraffin-embedded; FC, fold change; NA, not available.

### miRNA meta-signature of EOC

**Figure 1:**
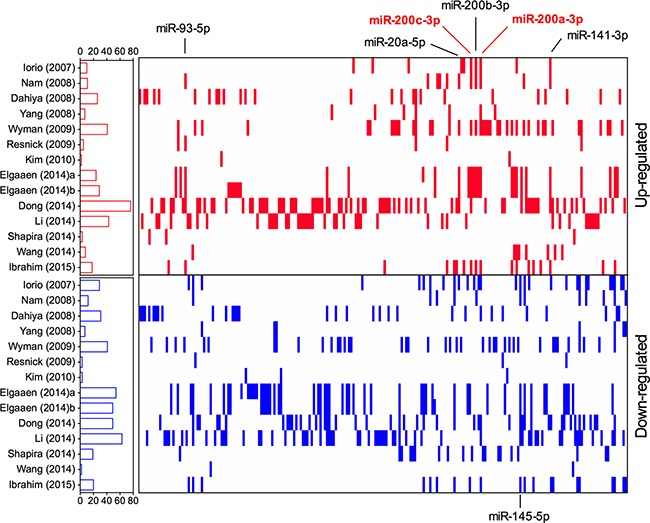
Distribution of miRNA alterations in EOC as reported in 14 primary miRNA profiling datasets Short red and blue vertical bars indicated upregulated and downregulated miRNAs, respectively. miRNAs are aligned according to miRBase release 21. The number of miRNAs in each study is graphically depicted on the left. The positions of EOC integrated-signature miRNAs have been marked.

In total, 203 significantly up-regulated miRNAs and 222 significantly down-regulated miRNAs were recorded, respectively. In Figure 1, we listed the upregulated (red vertical bars) and downregulated miRNAs (blue vertical bars) which were reported in at least one study. Among of them, 21 upregulated (10.3%, 21/203) and 39 downregulated (17.6%, 39/222) miRNAs were reported in more than 3 studies, respectively. Besides, there were 55 discordant alteration miRNAs which were found to be both up-regulated and down-regulated across the different studies. It should be pointed out that, when analyzed the lists of upregulated and downregulated miRNAs which were reported in more than 3 studies, we also found two discordant alteration miRNAs, miR-126-3p (reported 4 times as upregulated miRNA and 4 times as downregulated miRNA) and miR-29a-3p (reported 4 times as upregulated miRNA and 3 times as downregulated miRNA). These inconsistent data indicated a problematic inter-lab reproducibility of miRNA profiling studies.

**Figure 2:**
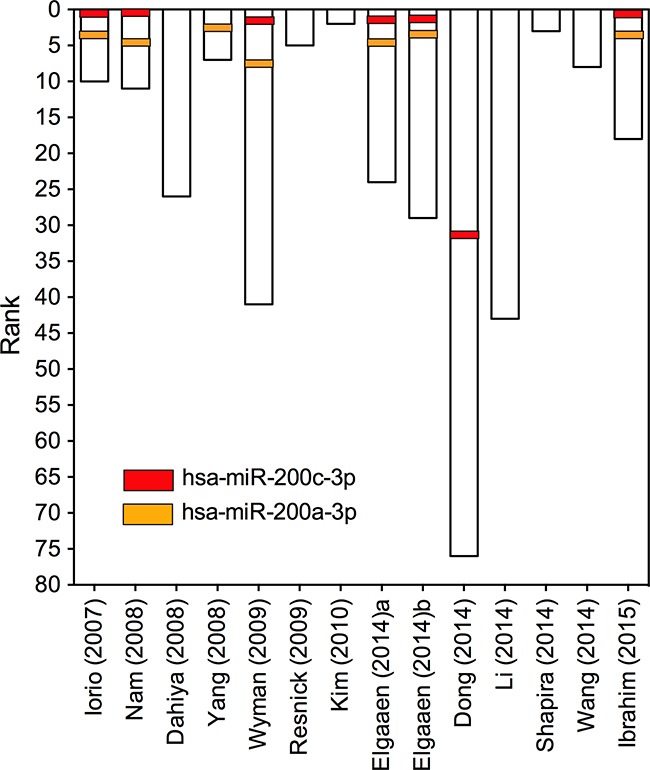
Ranks for miR-200a-3p and miR-200c-3p in 14 primary miRNA profiling datasets The ranks for miR-200a-3p and miR-200c-3p in each of the enrolled study were depicted. Each column represents one of the 14 primary miRNA profiling datasets. The rank of miRNAs in each study is graphically depicted on the left.

To resolve the differences that exist among studies, we performed a meta-analysis using a recent published robust rank aggregation method. We identified a statistically significant meta-signature of six upregulated miRNAs (miR-200c-3p, miR-200a-3p, miR-141-3p, miR-200b-3p, miR-93-5p and miR-20a-5p) and one downregulated miRNA (miR-145-5p) (Table 2). The direction of expression change of all above miRNAs was consistent across all enrolled studies, with *P*-value ranged from 3.37E-04 to 9.40E-09. Majority of the meta-signature miRNAs belong to the broadly conserved seed families. Intriguingly, the top four meta-signature upregulated miRNAs (miR-200c-3p, miR-200a-3p, miR-141-3p, and miR-200b-3p) belonged to the same seed family, miR-200abc/141/429 family, which might play an important role in carcinogenesis of EOC. Two miRNAs, miR-200c-3p and miR-200a-3p were significantly upregulated in EOC (Bonferroni-corrected *P*=1.94E-05 and1.88E-03, respectively), but no downregulated miRNA reached statistical significance after Bonferroni-correction. Both miR-200c-3p and miR-200a-3p were reported to be significantly upregulated in EOC tissues by majority of the studies (7/14) with relatively high rank scores (Figure 2), suggesting their potential values as biomarkers for diagnosis of EOC.

**Table 2:** Meta-signature miRNAs in ovarian cancer

microRNA	Chromosome	*P*-value	Corrected *P-*value	Studies	miRNA seed family	miRNA cluster
**Up-regulated**						
hsa-miR-200c-3p	12p13.31	9.40E-09	1.94E-05	7	miR-200abc/141/429 family	miR-200c/141 cluster
hsa-miR-200a-3p	1p36.33	9.09E-07	1.88E-03	7	miR-200abc/141/429 family	miR-200ab/429 cluster
hsa-miR-141-3p	12p13.31	4.28E-05	8.83E-02	6	miR-200abc/141/429 family	miR-200c/141 cluster
hsa-miR-200b-3p	1p36.33	1.02E-04	2.10E-01	6	miR-200abc/141/429 family	miR-200ab/429 cluster
hsa-miR-93-5p	7q22.1	1.85E-04	3.82E-01	6	miR-17/20ab/106ab/93 family	miR-106b-25 cluster
hsa-miR-20a-5p	13q31.3	1.90E-04	3.92E-01	5	miR-17/20ab/106ab/93 family	miR-17-92 cluster
**Down-regulated**						
hsa-miR-145-5p	5q32	3.37E-04	6.96E-01	7	miR-145 family	miR-143/145 cluster

### Diagnostic accuracy of miR-200a/c in EOC

To explore the diagnostic efficiencies of miR-200a-3p and miR-200c-3p in EOC, we next performed a diagnostic meta-analysis. Briefly, we searched all published clinical studies which simultaneously detected miR-200a-3p and miR-200c-3p levels in EOC. According to the search criteria, 5 independent studies retrieved from 4 articles [18, 29–31] were finally enrolled and the detailed information was listed in Table 3. Goodness of fit and bivariate normality analysis revealed that the random-effect bivariate model was robust for the calculation of the pooled estimates (Supplementary Figure S1 and S2). We further employed Deeks’ funnel plot asymmetry test to assess publication bias. The slope coefficient was associated with a *P* value of 0.61 and 0.41 for miR-200a-3p and miR-200c-3p, respectively (Supplementary Figure S3), suggesting no publication bias in following meta-analysis.

**Table 3:** Characteristics of studies included for diagnostic meta-analysis

Author (year)	Country	Ethnicity	Period	No. of sample (Case/Control)	Cancer subtypes	Sample source	Method
Nam (2008) [18]	Korea	Asian	NA	20/8	20 serous ovarian carcinomas.	Tissue	qRT-PCR
Kan (2012)a [30]	Australia	Caucasian	NA	28/28	28 serous epithelial ovarian cancer.	Serum	qRT-PCR
Kan (2012)b [30]	Australia	Caucasian	NA	28/28	28 serous epithelial ovarian cancer.	Serum	qRT-PCR
Zuberi (2015) [31]	India	Asian	2012/1-2014/10	52/18	36 mucinous, 17 serous, 8 papillary, 1 clear cell, 2 endometroid, 2 undifferentiated, 1 mixed epithelial ovarian cancer.	Serum	qRT-PCR
Ibrahim (2015) [29]	Malaysia	Asian	2006-2013	22/22	22 serous epithelial ovarian cancer.	Tissue	qRT-PCR

a Immortalized human ovarian surface epithelial cells were used as control.

b Benign tumors were used as control.

FFPE, formalin-fixed paraffin-embedded; FC, fold change; NA, not available.

**Figure 3:**
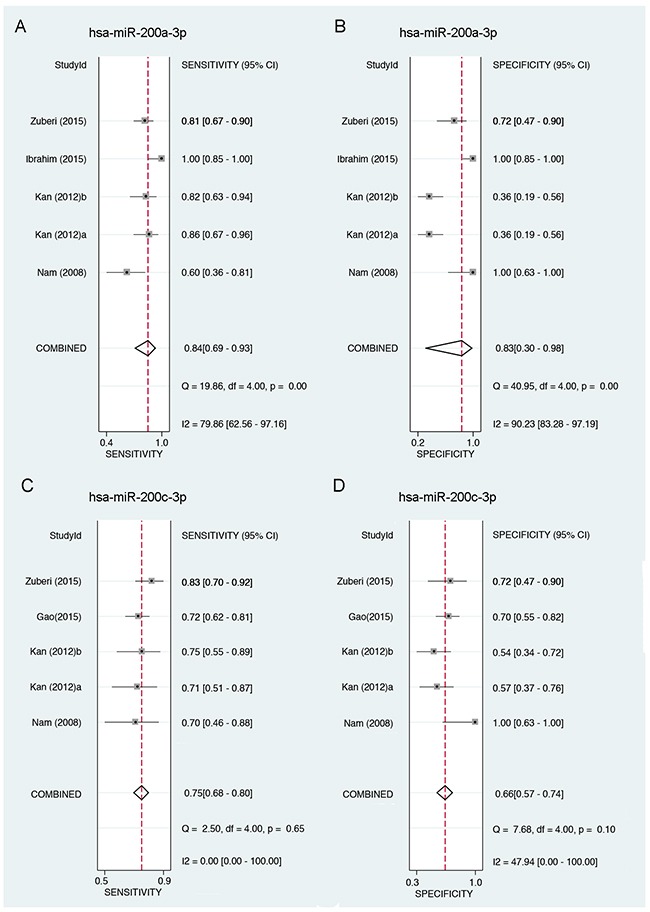
Forest plots showing the sensitivity and specificity of miR-200a/c in the diagnosis of EOC **A.** Forest plot showing the sensitivity of miR-200a-3p in the diagnosis of EOC. **B.** Forest plot showing the specificity of miR-200a-3p in the diagnosis of EOC. **C.** Forest plot showing the sensitivity of miR-200c-3p in the diagnosis of EOC. **D.** Forest plot showing the specificity of miR-200c-3p in the diagnosis of EOC. 95%CI: 95% of confidence interval; EOC: epithelial ovarian cancer.

**Figure 4:**
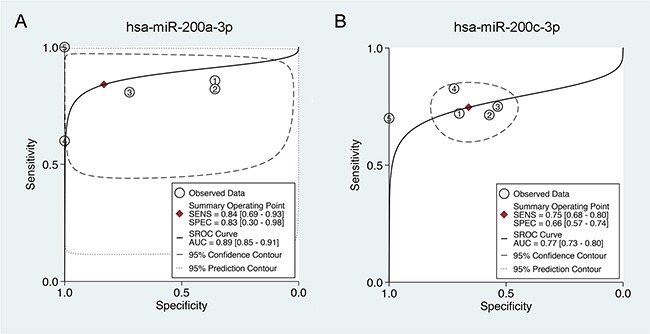
Summary receiver operating characteristic (SROC) curve of miR-200a/c in the diagnosis of EOC **A.** Summary receiver operating characteristic (SROC) curve of miR-200a-3p in the diagnosis of EOC. **B.** Summary receiver operating characteristic (SROC) curve of miR-200c-3p in the diagnosis of EOC. AUC: area under curve; SROC curve, summary receiver operator curve; SENS: sensitivity; SPEC: specificity; EOC: epithelial ovarian cancer.

As shown in Figure 3, miR-200a-3p showed a relatively high sensitivity (ranged from 0.60 to 1.0 among different studies, while a pooled sensitivity reached 0.84) (Figure 3A) and a moderate specificity (ranged from 0.36 to 1.0 among different studies, while a pooled specificity reached 0.83) in EOC diagnosis (Figure 3B). As for miR-200c-3p, similar results are generated: the diagnostic sensitivity among different studies ranged from 0.70 to 0.83 (with a pooled sensitivity 0.75) (Figure 3C), while the specificity among different studies ranged from 0.54 to 1.0 (with a pooled specificity 0.66) (Figure 3D). SROC curves were also depicted to evaluate the diagnostic capacities of miR-200c-3p (Figure 4A) and miR-200a-3p (Figure 4B). The summary AUC for miR-200a-3p was 0.89 (95%CI, 0.85-0.91) and 0.77 for miR-200c-3p (95%CI, 0.73-0.80), respectively, both of which showed relatively high diagnostic efficiencies for EOC (Figure 4).

### The impact of miR-200a/c on cellular pathways and biological processes

**Figure 5:**
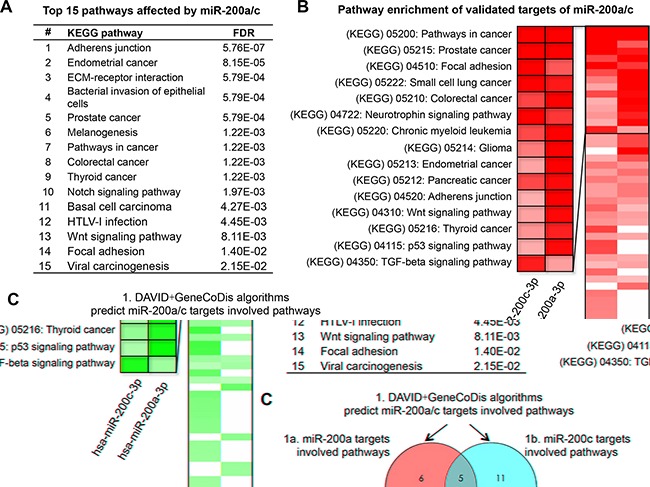
miR-200a/c target prediction and pathway enrichment analysis **A.** The top 15 saturated pathways affected by miR-200a/c. **B.** Heatmap of the pathway enrichment of validated miR-200a/c target genes. Rows: pathways; Columns: genes. Range of colors (deep red to white) shows the range of expression values (high to low). **C.** Schematic of pathway prediction results by web tools including DAVID + GeneCodis algorithms and DIANA mirpath algorithm.

Experimentally validated targets from miRTarBase (http://mirtarbase.mbc.nctu.edu.tw/) were extracted to elucidate the biological function of miR-200a-3p and miR-200c-3p. Pathway enrichment analysis of validated targets of miRNAs was conducted using two different algorithms, DAVID (https://david.ncifcrf.gov/) and GeneCodis (http://genecodis.dacya.ucm.es/). By combining the prediction results from DAVID and GeneCodis, we found 20 KEGG pathways might be affected by dysregulated miR-200a-3p and 19 pathways for miR-200c-3p (Supplementary Table S1). As shown in Figure 5B, the most common pathways that affect by both miR-200a-3p and miR-200c-3p were associated with multiple cancers and cellular adhesion-related pathways (including KEGG_04510 Focal adhesion and KEGG_04520 Adherens junction). When employing the miRPath algorithm (http://www.microrna.gr/miRPathv2) to calculate the combinatorial effect of miR-200a-3p and miR-200c-3p in cellular pathway, our results showed that miR-200a/c might affect cellular adhesion-related pathways (KEGG_04520 Adherens junction with a FDR of 5.76E-07; KEGG_04510 Focal adhesion with a FDR of 1.40E-02) (Figure 5A and Supplementary Table S2). Moreover, KEGG_04510 Focal adhesion, as one of the most frequently predicted pathways by multiple algorithms (Figure 5C), was key component in the epithelial-mesenchymal transition (EMT) and was commonly recognized as an important event and the initiating stage for tumor invasion and metastasis.

**Figure 6:**
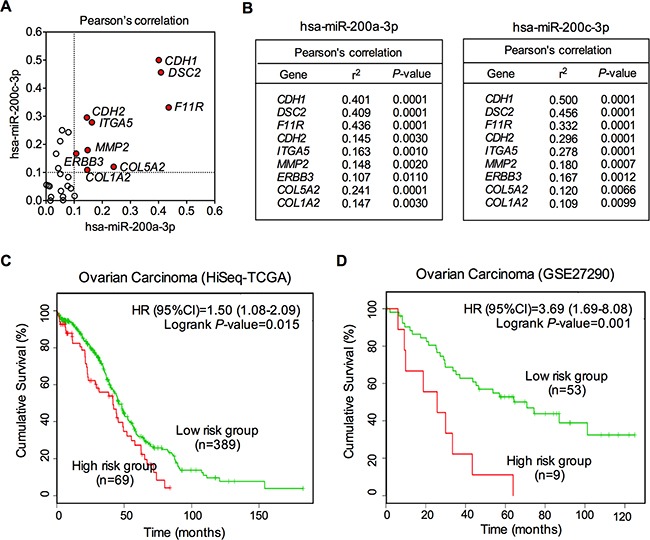
Correlation of miR-200a/c with cellular adhesion-related genes **A.** Schematic of Pearson's correlation of miR-200a/c with cellular adhesion-related genes. Genes with an r^2^ >0.1 were shown as red dots. **B.** Pearson's correlation of miR-200a/c with cellular adhesion-related genes. The r^2^ and P values for each of the top 9 genes were demonstrated for miR-200a and miR-200c. **C.** Kaplan–Meier analysis of overall survival for patients with different miR-200a/c expression levels. Data were drawn from two independent cohort studies (HiSeq-TCGA and GSE27290). The red lines represent the high risk groups, and the green lines represent the low risk groups.

To better explore the relationship between miR-200a/c and cellular adhesion process, we performed expression correlation analysis based on miRNA and mRNA expression profiles in 60 human cancer cell lines (the National Cancer Institute's NCI-60 cell line panel). All raw data were downloaded from GEO (www.ncbi.nlm.-nih.gov/geo/). GSE5846 was mRNA expression profile including 22207 genes, and GSE26375 was miRNA expression profile including 422 miRNAs. The cellular adhesion-related gene list was generated from SABiosciences system (http://www.sabiosciences.com/). Among 29 cellular adhesion-related genes, 9 genes (*CDH1*, *DSC2*, *F11R*, *CDH2*, *ITGA5*, *MMP2*, *ERBB3*, *COL5A2* and *COL1A2*) were associated with both miR-200a-3p and miR-200c-3p (all *P*<0.01, Figure 6A). Interestingly, the top three genes that related to miR-200a-3p and miR-200c-3p were consistent (Figure 6B). They were *CDH1*, *DSC2* and *F11R*, all of which were key regulatory genes in EMT process. Their encoded proteins, E-cadherin, desmocollin 2 and junctional adhesion molecule-A (JAM-A) had been widely accepted as prognostic biomarkers for multiple epithelial cancers, including ovarian cancer [32–34]. All above data suggested that miR-200a/c might be involved in cellular adhesion and EMT process, and associated with clinical outcome.

### The associated between dysregulated miR-200a/c and clinical outcome

Kaplan-Meier survival analysis was used to analyze the association between miR-200a/c expression and overall survival in EOC patients. To minimize the bias caused by chance, clinical data were obtained from two independent datasets for following survival analysis. These two datasets employed completely different detection platforms, Illumina Next-Generation Sequencing platform (HiSeq-TCGA dataset, n=458) and Agilent-015508 Human miRNA Microarray platform (GSE27290 dataset, n=62). To reduce inter-lab variability, we performed survival analysis of these two datasets separately. By using SurvMicro, a web-based tool for assessment of miRNA-based prognostic signatures by multivariate survival analysis (http://bioinformatica.mty.itesm.mx/SurvMicro), we divided the ovarian patients into two groups, high risk and low risk groups. As shown in Figure 6C and 6D, miR-200a/c was significantly associated with overall survival of EOC in both two datasets, with log rank *P*-values being 0.015 and 0.001, respectively (Figure 6C and 6D). The Cox regression model demonstrated that EOC patients in high risk groups showed lower cumulative survival rates, compared with those in low risk groups (Figure 6C and 6D). The HR values were 1.50 (95%CI, 1.08-2.09) and 3.69 (95%CI, 1.69-8.08), respectively, suggesting a potential value of dysregulated miR-200a/c in survival prediction for EOC patients.

## DISCUSSION

There is an increasing body of evidence supporting the crucial roles of microRNAs in the carcinogenesis and progression of EOC [35–37]. However, consistent conclusion rarely yielded from miRNA expression profiling studies among diverse studies, mainly due to the differences in measurement platforms, lab protocols, tumor histological characteristics and heterogeneity, as well as incomparable gene expression levels rendered by small sample sizes [38, 39]. Efforts have been made to compensate for these shortcomings, including the performances of systematic review or meta-analysis. Unfortunately, no favorable results have yet been generated for the lack of cross-platform standardization of miRNA detecting technologies as well as/or the unavailability of raw data.

Here in our study, we applied the RRA method, which was specifically designed to compare several ranked gene lists as well as identify commonly overlapping genes. In RRA analysis, each miRNA profiled would be re-ranked and its significance re-determined, as well as calculated to generate a true combined *P* value, and these efforts together would overcome the drawbacks of traditional systematic review or meta-analysis. By this RRA approach, we enrolled 14 prioritized miRNA lists detected from a total of 519 EOC and 248 noncancerous tissue samples, and finally identified an integrated-signature of six upregulated and one downregulated EOC-specific miRNAs. Most integrated-signature miRNAs have been reported to be involved in the carcinogenesis and progression.

The miR-200 family was depicted as versatile players in various cancer types, including ovarian cancer [40–43]. This family consists of 5 members, namely miR-200a, -200b, -200c, -141, and -429. Up-regulation of miR-200a, -141, -200c and -200b has been reported in a study that compared expression profiles of normal ovarian tissue and ovarian cancer to determine a miRNA signature for ovarian cancer [17]. Another study also found elevated miR-200a expression in ovarian tumor tissues [44]. These results, together with others, indicated the possibility that elevated miR-200 family expression may be a significant characteristic of ovarian cancers compared to their non-cancerous counterparts. In the present study, after a Bonferroni-correction which was applied to control for Type I error rate, miR-200c-3p and miR-200a-3p remained the most significantly upregulated miRNA in EOC, suggesting their critical roles and diagnostic values for patients with EOC. In the following validation part, we therefore performed a diagnostic meta-analysis which enrolled all the studies available that detected miR-200a and miR-200c simultaneously in EOC samples. Intriguingly, both miR-200a and miR-200c demonstrated favorable sensitivity and specificity in EOC diagnosis. The summary AUCs for miR-200a-3p and miR-200c-3p were 0.89 and 0.77, respectively, both of which showed relatively high diagnostic efficiencies for EOC.

To explore the underlying molecular relationship between miR-200a/c and EOC, we furthermore performed pathway enrichment analysis. Pathway enrichment of experimentally validated targets suggested that the miR-200a/c was involved in cellular adhesion-related pathways. Experimental validation based on 60 human cancer cell lines confirmed a strong correlation between miR-200a/c and key cellular adhesion-related genes. Admittedly, the role of miR-200 in cellular adhesion process has been frequently depicted in many studies. Davalos V et al observed that the miR-200 family was highly expressed within epithelial cells and was involved in maintaining epithelial integrity [45]. The miR-200 family miRNAs have also been found to downregulate ZEB1 and ZEB2 expression, both of which were key transcription factors in EMT mediation, and effectively upregulate the cellular E-cadherin level to maintain a cell in a more epithelial-like status [46–49]. Since cellular adhesion is the initial process during cancer cell EMT and metastasis, we then explored the values of miR-200a/c in predicting clinical outcome. Kaplan-Meier survival analysis based on two independent cohorts revealed a significantly correlation between miR-200a/c levels and patients’ overall survival, providing solid foundation for future efforts to interpret the impact of miR-200a/c on EOC survival. Drugs targeting miR-200a/c might provide novel options for treatment against EOC in future clinical practice. The jury must refrain from drawing a conclusion until multi-center, well-performed studies confirm or refuse our findings.

In conclusion, we report here that miR-200a-3p and miR-200c-3p were the two mostly dysregulated miRNAs in EOC. We provided evidence for the key role of miR-200a/c in the cellular adhesion process of EOC. Our findings warrant further clinical studies to validate miR-200a/c as a biomarker for the diagnosis, treatment, as well as prognosis prediction of EOC.

## MATERIALS AND METHODS

### Literature search

A systematic literature search was performed for the identification of ovarian cancer miRNA expression profiling studies that had been published prior to December 31^st^, 2015 using a two-step search strategy. First, we performed a web-based search in Pubmed (www.ncbi.nlm.nih.gov/pubmed), Embase (www.embase.com/) and Web of Knowledge (http://apps.webofknowledge.com/) databases using search term combination of (mirna* OR microrna* OR mir-*) AND profil* AND ((ovary AND (cancer* OR tumor* OR tumour* OR carcinoma)) OR (ovarian* AND (cancer* OR tumor* OR tumour* OR carcinoma)). To perform a comprehensive retrieval, searching in ArrayExpress (www.ebi.ac.uk/arrayexpress) and Gene Expression Omnibus (GEO, www.ncbi.nlm.-nih.gov/geo/) were also performed. Second, citations of all relevant and existing studies were also screened through a manual search for further identification of potential relevant studies. Authors were contacted when the miRNA lists were not available in the publications.

### Inclusion/exclusion criteria

Only original experimental articles published in English language were included. Abstracts were screened carefully and full texts of relevant potential abstracts were evaluated. Studies that use a high-throughput miRNA expression profiling method such as second-generation sequencing or microarray-based methods in ovarian cancer tissue versus adjacent non-cancerous ovarian tissue were included. Those studies employed polymerase chain reaction (PCR) methods that designed for parallel quantification of large number of miRNAs (96- or 384-wellmicroplates) were also included. Meanwhile, exclusion criteria include: 1) studies that used only cell lines; 2) studies that preselected individual candidate miRNA genes; and 3) studies that profiled different clinical or histologic subtypes without non-cancerous control tissues included.

### Data extraction and RRA analysis

Lists of statistically differentially expressed miRNAs were extracted from the included studies. All miRNA names were standardized according to miRBase version 21 (http://www.mirbase.org/). Viral miRNAs and non-miRNA probes were excluded. Pre-miRNAs, listed in some of the studies were also included in the analyses once the precursor names were standardized. A novel RRA method implemented as an R package “Robust Rank Aggreg” was used to identify miRNAs that were ranked consistently better than expected by chance [15]. This method detects miRNAs that are ranked consistently better than expected under null hypothesis of uncorrelated inputs and assigns a *P*-value for each miRNA. All the RRA tools used in the present study are freely available in the comprehensive R Archive Network website (http://cran.r-project.org/).

### Diagnostic meta-analysis

To explore the diagnostic value of meta-signature miRNA in ovarian cancer patients, we conducted a diagnostic meta-analysis by the STATA12.0 software. A literature search for studies was conducted among Pubmed (www.ncbi.nlm.nih.gov/pubmed), Embase (www.embase.com/) and Web of Knowledge (http://apps.webofknowledge.com/) databases. The search terms we used were as follows: (microrna-200 OR mirna-200 OR mir-200) AND ((ovary AND (cancer* OR tumor* OR tumour* OR carcinoma)) OR (ovarian* AND (cancer* OR tumor* OR tumour* OR carcinoma)) AND (sensitivity OR specificity). Eligible data from included studies were extracted including basic characteristics of studies and diagnostic data [sensitivity, specificity, area under the curve (AUC), true positive (TP), true negative (TN), false positive (FP), and false negative (FN)] of each studies. To evaluate diagnostic effects, bivariate meta-analysis models were employed to calculate the pooled sensitivity, specificity and AUC. The summary receiver operator characteristic (ROC) curve was then fitted to above pooled sensitivity and specificity of each study. To explore whether there was publication bias in our included studies, we performed Deek's funnel plot. If the *P* value was less than 0.10, it means that publication bias is significant.

### Validated targets of miRNAs

To avoid incorrectly miRNA functional annotation which usually caused by erroneous target predictions, in the present study, we therefore used validated targets of miRNAs for further analysis. The validated targets of meta-signature miRNAs were obtained using databases miRTarBase (http://mirtarbase.mbc.nctu.edu.tw/). We only included validated targets with “strong evidence”. The “strong evidence” means that the miRNA-target relationship was validated by at least one of the following experimental methods, reporter assay, western blot or qPCR.

### Pathway enrichment analysis

Enrichment analysis for Kyoto Encyclopedia of Genes and Genomes (KEGG) was performed with DAVID (https://david.ncifcrf.gov/) and GeneCodis tools (http://genecodis.dacya.ucm.es/). The potential targets of each miRNA were used as input and false discovery rate (FDR)-corrected *P*-values were visualized as a heatmap. Clustering of the heatmap was based on Pearson correlation and average linkage. Moreover, the combinatorial effects of multiple miRNAs in pathways were predicted using “miRPath” algorithm with the DIANA tools (http://www.microrna.gr/miRPathv2).

## SUPPLEMENTARY MATERIALS FIGURES AND TABLES




