# Supplementary material for: miRNA-200a/c as potential biomarker in epithelial ovarian cancer (EOC): evidence based on miRNA meta-signature and clinical investigations
Source: Oncotarget. 2016 Nov 7;7(49):81621–33. doi: 10.18632/oncotarget.13154 (PMC5348417; doi:10.18632/oncotarget.13154)
Supplement: Supplementary file 1 [file oncotarget-07-81621-s001.pdf]

miRNA-200a/c as potential biomarker in epithelial ovarian cancer (EOC): evidence based on miRNA meta-signature and clinical investigations

SUPPLEMENTARY FIGURES AND TABLES

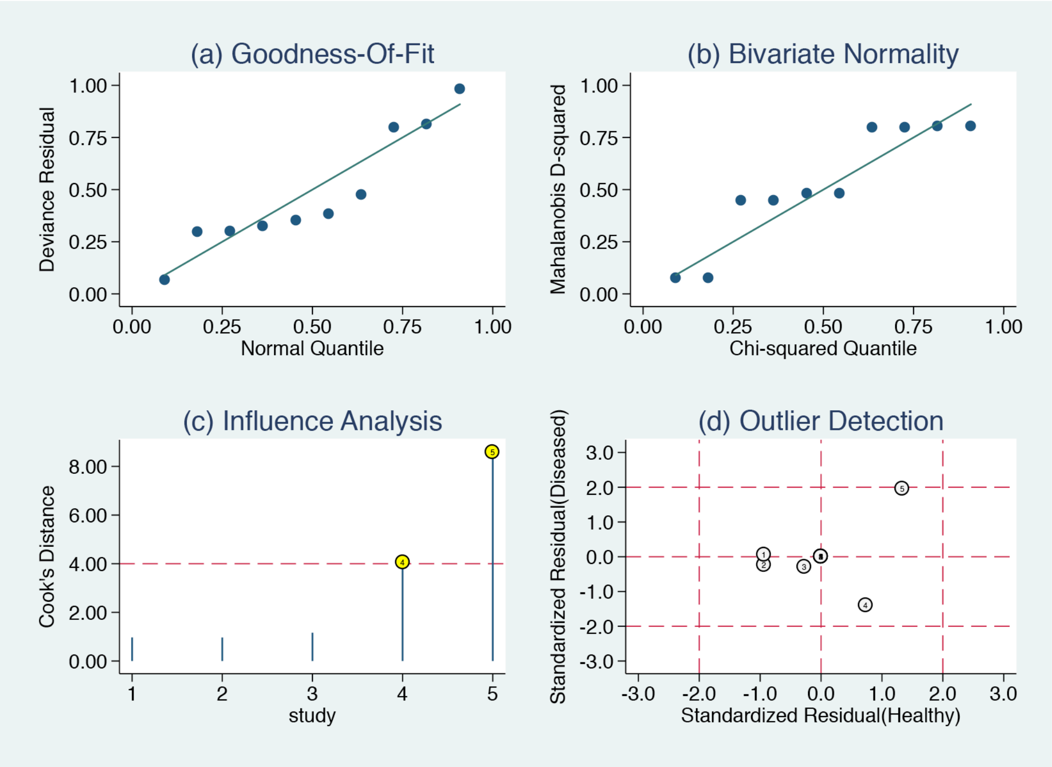

Supplementary Figure S1: Sensitivity analysis of miR-200a in EOC. A. goodness-of-fit; **B.** bivariate normality; **C.** influence analysis; **D.** outlier detection.

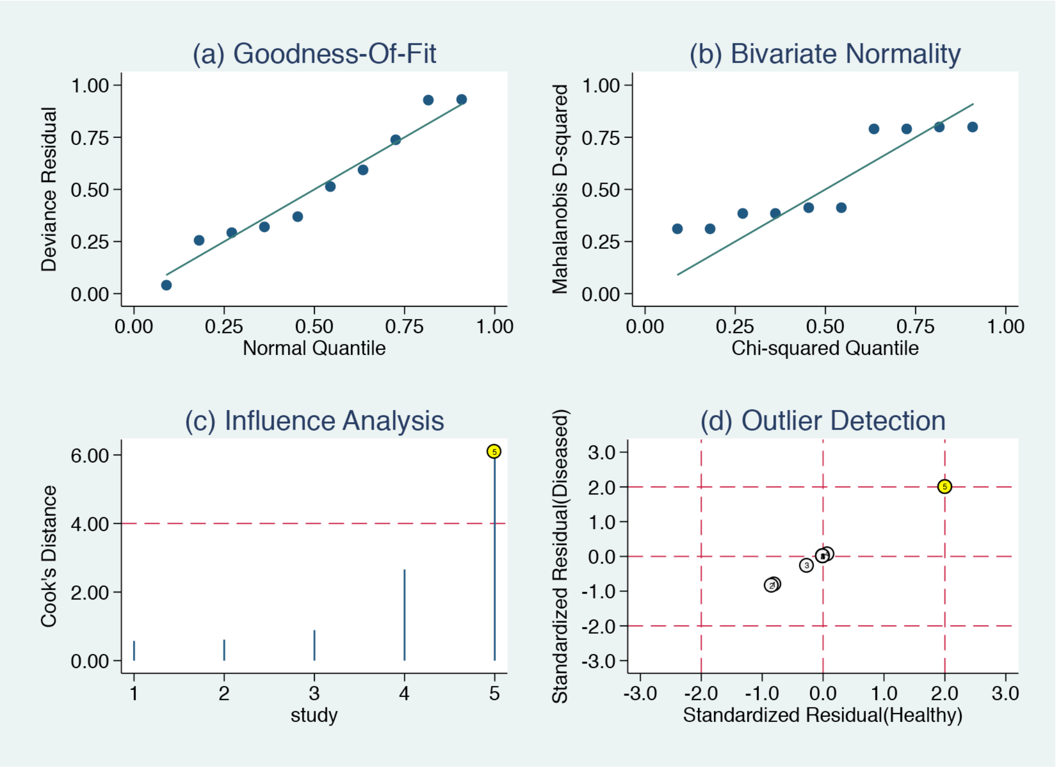

Supplementary Figure S2: Sensitivity analysis of miR-200c in EOC. A. goodness-of-fit; **B.** bivariate normality; **C.** influence analysis; **D.** outlier detection.

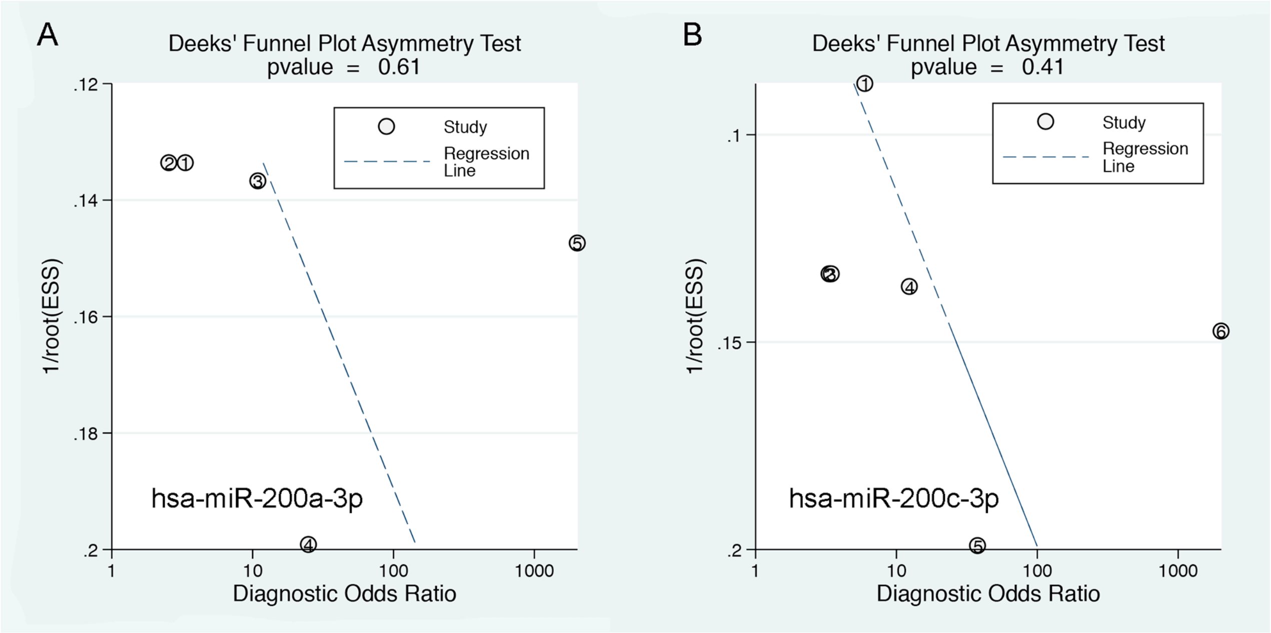

Supplementary Figure S3: Funnel graph for the assessment of potential publication bias for studies ofmiR-200a A. and miR-200c **B.**

Supplementary Table S1: The combinatorial effect of miR-200a-3p and miR-200c-3p in pathways predicted by DAVID and GeneCoDis algorithms.

See Supplementary File 1

Supplementary Table S2: The combinatorial effect of miR-200a-3p and miR-200c-3p in pathways predicted by DIANA miRPath algorithm

#	KEGG pathway	FDR
1	Adherens junction	5.76E-07
2	Endometrial cancer	8.15E-05
3	ECM-receptor interaction	5.79E-04
4	Bacterial invasion of epithelial cells	5.79E-04
5	Prostate cancer	5.79E-04
6	Melanogenesis	1.22E-03
7	Pathways in cancer	1.22E-03
8	Colorectal cancer	1.22E-03
9	Thyroid cancer	1.22E-03
10	Notch signaling pathway	1.97E-03
11	Basal cell carcinoma	4.27E-03
12	HTLV-I infection	4.45E-03
13	Wnt signaling pathway	8.11E-03
14	Focal adhesion	1.40E-02
15	Viral carcinogenesis	2.15E-02
16	Arrhythmogenic right ventricular cardiomyopathy (ARVC)	2.64E-02
17	Leukocyte transendothelial migration	3.03E-02
18	HIF-1 signaling pathway	3.03E-02
