# Supplementary material for: miRNA-200a/c as potential biomarker in epithelial ovarian cancer (EOC): evidence based on miRNA meta-signature and clinical investigations
Source: Oncotarget. 2016 Nov 7;7(49):81621–33. doi: 10.18632/oncotarget.13154 (PMC5348417; doi:10.18632/oncotarget.13154)
Supplement: Supplementary file 2 [file oncotarget-07-81621-s002.docx]

**Table S1 The combinatorial effect of miR-200a-3p and miR-200c-3p in pathways predicted by DAVID and GeneCoDis algorithms**

| **#** | **KEGG pathway** | **DAVID** | **GeneCoDis** |
| --- | --- | --- | --- |
| **hsa-miR-200a-3p** | | | |
| 1 | Adherens junction | 7.70E-04 | 5.36E-07 |
| 2 | Basal cell carcinoma | 4.70E-02 | 2.43E-04 |
| 3 | Cell cycle | 4.40E-03 | 6.13E-06 |
| 4 | Chronic myeloid leukemia | 8.10E-04 | 4.92E-07 |
| 5 | Colorectal cancer | 1.60E-04 | 3.75E-07 |
| 6 | Endometrial cancer | 2.80E-04 | 2.03E-07 |
| 7 | ErbB signaling pathway | 9.20E-02 | 1.38E-02 |
| 8 | Focal adhesion | 8.00E-02 | 5.75E-04 |
| 9 | Glioma | 4.90E-04 | 3.26E-07 |
| 10 | MAPK signaling pathway | 1.50E-01 | 1.54E-03 |
| 11 | Melanoma | 7.10E-02 | 4.70E-04 |
| 12 | Neurotrophin signaling pathway | 2.90E-02 | 1.18E-04 |
| 13 | Non-small cell lung cancer | 4.80E-02 | 2.45E-04 |
| 14 | p53 signaling pathway | 7.30E-03 | 1.51E-05 |
| 15 | Pancreatic cancer | 7.90E-03 | 1.55E-05 |
| 16 | Pathways in cancer | 2.20E-04 | 3.24E-08 |
| 17 | Prostate cancer | 1.40E-04 | 7.40E-08 |
| 18 | Small cell lung cancer | 1.10E-02 | 3.08E-05 |
| 19 | Thyroid cancer | 1.70E-02 | 4.27E-05 |
| 20 | Wnt signaling pathway | 7.90E-03 | 1.40E-05 |
| **hsa-miR-200c-3p** | | | |
| 1 | Acute myeloid leukemia | 2.80E-01 | 2.38E-03 |
| 2 | Axon guidance | 1.20E-01 | 2.05E-04 |
| 3 | Bladder cancer | 2.10E-01 | 1.10E-03 |
| 4 | Chemokine signaling pathway | 2.40E-01 | 8.60E-04 |
| 5 | Chronic myeloid leukemia | 1.20E-01 | 2.86E-04 |
| 6 | Dorso-ventral axis formation | 1.40E-01 | 2.80E-04 |
| 7 | Focal adhesion | 8.30E-04 | 4.96E-09 |
| 8 | Glioma | 2.90E-01 | 2.84E-03 |
| 9 | Neurotrophin signaling pathway | 6.30E-03 | 1.04E-06 |
| 10 | NOD-like receptor signaling pathway | 3.00E-01 | 2.48E-03 |
| 11 | Pancreatic cancer | 1.20E-01 | 2.65E-04 |
| 12 | Pathogenic Escherichia coli infection | 2.90E-01 | 2.28E-03 |
| 13 | Pathways in cancer | 5.90E-04 | 4.40E-09 |
| 14 | Prostate cancer | 6.50E-03 | 2.01E-06 |
| 15 | Regulation of actin cytoskeleton | 1.20E-01 | 2.34E-04 |
| 16 | Renal cell carcinoma | 3.20E-01 | 3.47E-03 |
| 17 | RIG-I-like receptor signaling pathway | 3.20E-01 | 3.45E-03 |
| 18 | Small cell lung cancer | 6.20E-03 | 2.04E-06 |
| 19 | TGF-beta signaling pathway | 4.30E-02 | 3.70E-05 |
